# A novel Arabidopsis pathosystem reveals cooperation of multiple hormonal response-pathways in host resistance against the global crop destroyer *Macrophomina phaseolina*

**DOI:** 10.1038/s41598-019-56401-2

**Published:** 2019-12-27

**Authors:** Mercedes M. Schroeder, Yan Lai, Miwa Shirai, Natalie Alsalek, Tokuji Tsuchiya, Philip Roberts, Thomas Eulgem

**Affiliations:** 1 Center for Plant Cell Biology, Institute of Integrative Genome Biology, Department of Botany and Plant Sciences, University of California, Riverside, Riverside, California, United States of America; 2 College of Life Sciences, Fujian Agricultural and Forestry University, Fuzhou, Fujian, China; 3 School of Pharmacy, University of California, San Francisco, San Francisco, California, United States of America; 4 College of Bioresource Sciences, Nihon University, Kanagawa, Japan; 5 Institute of Integrative Genome Biology, Department of Nematology, University of California, Riverside, Riverside, California, United States of America

**Keywords:** Transcriptomics, Microbe

## Abstract

Dubbed as a “global destroyer of crops”, the soil-borne fungus *Macrophomina phaseolina* (*Mp*) infects more than 500 plant species including many economically important cash crops. Host defenses against infection by this pathogen are poorly understood. We established interactions between *Mp* and *Arabidopsis thaliana* (Arabidopsis) as a model system to quantitatively assess host factors affecting the outcome of *Mp* infections. Using agar plate-based infection assays with different Arabidopsis genotypes, we found signaling mechanisms dependent on the plant hormones ethylene, jasmonic acid and salicylic acid to control host defense against this pathogen. By profiling host transcripts in *Mp*-infected roots of the wild-type Arabidopsis accession Col-0 and *ein2/jar1*, an ethylene/jasmonic acid-signaling deficient mutant that exhibits enhanced susceptibility to this pathogen, we identified hundreds of genes potentially contributing to a diverse array of defense responses, which seem coordinated by complex interplay between multiple hormonal response-pathways. Our results establish *Mp*/Arabidopsis interactions as a useful model pathosystem, allowing for application of the vast genomics-related resources of this versatile model plant to the systematic investigation of previously understudied host defenses against a major crop plant pathogen.

## Introduction

The broad host-spectrum pathogen *Macrophomina phaseolina* (*Mp*) is a devastating soil-borne fungus that infects more than 500 plant species^1–5^. Many of these hosts are economically important crop plants including maize, soybean, canola, cotton, peanut, sunflower and sugar cane^5–10^. *Mp* is found throughout the world^11–15^, notably in warmer regions where crop diseases caused by the pathogen are typically associated with drought and heat stress^16^. Increasing numbers of first reports of *Mp*-caused crop diseases^6,9,12^ combined with the possibility of global warming effects benefitting the spread of this pathogen through stressed hosts makes the need to effectively combat this pathogen imperative.

*Mp* forms mycelia with microsclerotia imbedded in the hyphae^17^. A microsclerotium is an aggregation of 50–200 hyphal cells that form a compact mass and is generally brown to black in color when fully formed. Microsclerotia are asexual (non-sporic) propagation structures that may remain dormant for extended periods of time (depending on environmental conditions) prior to germination. Hyphae extend from microsclerotia, typically form appressoria upon contact with host plant tissues and penetrate plant dermal cells growing intra- and inter-cellularly in roots and other tissues^18,19^. *Mp* hyphae produce cell wall degrading enzymes (CWDEs) and phytotoxins^20^ and typically colonize vascular tissue resulting in plant wilting and often death^17,21^. Although mainly described as a necrotroph, *Mp* appears, in some cases, to exhibit a biotrophic phase early in its infection cycle^22^, and may be more correctly referred to as a hemibiotroph.

*Mp* microsclerotia can persist in soil for weeks to several years and are extremely difficult to eradicate^23,24^. Methods of *Mp* management include soil solarization, soil fumigation, no-tilling, crop rotation, flooding, fungicide and other soil amendments (including bio-agents and chemical additives) and seed treatments but the reduction in pathogen development is often only partial^25–31^. In addition, fungicidal treatments pose environmental health risks^8^. Field observations have suggested that *Mp* infections are rising because of restriction of the use of methyl bromide^27,32,33^ and alternative methods for *Mp* eradication are urgently sought.

Recent research with crop plants has contributed valuable information using *Mp*-susceptible-versus-resistant variety phenotyping^19,34^ and genetic studies^35–37^. This ongoing work is conducted in the field, greenhouse and laboratory. Semi-*in vitro* assays, such as those conducted by Bressano and coworkers^38^ and Chowdhury and coworkers^19^, have been utilized to observe the *Mp* infection process in the roots of certain crop plants. The Bressano group examined early infection of *Mp* hyphae in soybean roots^38^ while Chowdhury and coworkers observed *Mp* behavior in the vicinity, on the surface and inside sesame tissues and were able to quantify microsclerotia in sesame roots^19^.

Major strides to identify the specific genetic regulatory mechanisms involved in conferring resistance are still needed and the design of innovative strategies for efficient protection of crops against *Mp*-caused diseases requires detailed and comprehensive knowledge of host immune responses directed against this pathogen. The model plant *Arabidopsis thaliana* (Arabidopsis) has been a key research tool, both to gain information about plant defense and to analyze the infection process of a compatible pathogen^39,40^. It is unparalleled in its stable genetic transformation capability among multicellular organisms^41^. Its genome was fully sequenced almost two decades ago, large sets of Arabidopsis mutants with well-characterized defects in processes mediating immunity are available and valuable genomics-related resources have been developed and extensively used by the scientific community for over 20 years. As a result, an extensive body of knowledge on the molecular genetics, biochemistry and physiology of the immune system of this organism has accumulated. Various types of immune receptors, signaling components and transcription factors have been identified as important for pathogen defense in Arabidopsis^42–44^. Arabidopsis immune responses have further been found to be controlled by the defense hormones salicylic acid (SA), ethylene (ET) and jasmonic acid (JA). While SA is known to mainly mediate host immunity against pathogens with biotrophic lifestyles, ET and JA mediate protection against necrotrophic pathogens and herbivorous insects^45,46^. Surprisingly, to our knowledge, quantitative studies on interactions of Arabidopsis with *Mp* have not been reported in research articles and this versatile plant model system has not been deeply exploited for studies on host defense against *Mp*.

Here we report on a semi-*in vitro* assay system to study Arabidopsis/*Mp* interactions. Our assays allow for accurate quantitative assessment of *Mp* biomass growth in Arabidopsis roots and the extent of host disease symptoms in aerial plant parts. We found Arabidopsis mutants compromised in ET, JA and/or SA signaling to exhibit enhanced susceptibility to *Mp*. This effect was particularly robust in the ET/JA deficient *ein2/jar1* double-mutant. Profiling *Mp*-induced transcriptional responses in this line and its parental wild type background, Col-0, by RNA-seq, linked transcriptional up-regulation of multiple known JA, ET and SA response-regulators to immunity against this pathogen. Results described here will serve as a basis for more extensive systematic studies on *Mp* defense responses in the Arabidopsis model system and provide candidate genes to be further tested for their contribution to *Mp* protection in Arabidopsis and in agricultural crop plants.

## Results

### A quantitative assay system for *Mp*-infected Arabidopsis roots

**Figure 1:**
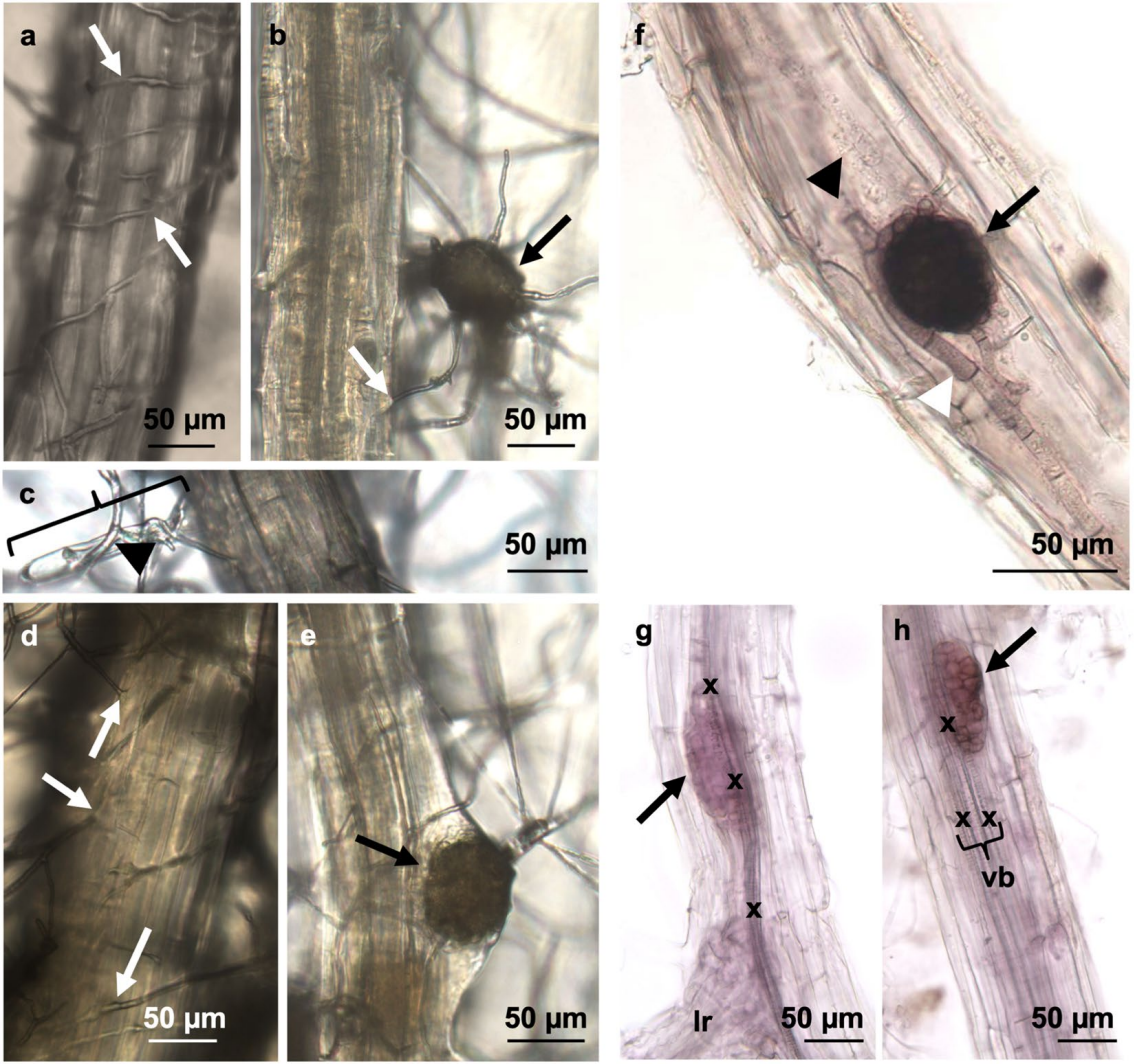
*Mp* infected Arabidopsis root tissue at 24 and 48 hours post-contact (hpc). (**A**,**D**) *Mp* hyphae surrounded and penetrated Arabidopsis roots at 24 (**A**) and 48 (**D**) hpc. (**B**) *Mp* microscerotium (black arrow), nearly in the microscopic focal plane with the root, shows relative size reference. (**C**) Bracket indicates a single root hair surrounded by hyphae at 24 hpc. (**E**) Microscerotia were forming inside root tissue at 48 hpc. (**F**) The black arrowhead points to hyphae emanating from the microsclerotium. The white arrowhead points to mature hyphae. (**G**,**H**) Acid fuchsin stained *Mp*-infected Arabidopsis roots. *Mp* microsclerotia began to form as early as 48 hpc throughout root tissue and were often associated with vascular tissue. White arrows point to locations of hyphal insertion into root tissue. Black arrows point to microsclerotia. Bracket indicates vascular bundle, vb; vascular bundle, x; xylem cells, lr; lateral root.

A fundamental step in the infection of plants by *Mp* is the establishment of hyphae and microsclerotia in plant tissue. We observed this process in *Mp*/Arabidopsis interactions on agar plates. *Mp* microsclerotia added to ½ MS agar generate hyphae that grow throughout the agar, forming more microsclerotia as *Mp* grows. When Arabidopsis seedlings are laid down upon the *Mp*-laden agar infection plate, hyphae on the agar surface contact the roots and grow toward them, surrounding and penetrating the root tissue (Fig. 1A,D). Microsclerotia can be seen forming within the roots as early as 48 hours post contact (hpc) in Arabidopsis (Fig. 1E).

The fact that critical events of the *Mp* infection process occur in Arabidopsis roots under controlled conditions on agar enables the design of plate-based infection protocols to accurately quantify levels of host susceptibility (Supplementary Fig. S1). Here, Arabidopsis seeds were sown on ½ MS agar plates (Supplementary Fig. S1A) and allowed to grow for ten days (Supplementary Fig. S1C) while *Mp* microsclerotia were added to separate plates (Supplementary Fig. S1B) and grown for six days (Supplementary Fig. S1D). The ten-day-old seedlings were transferred to the six-day-old *Mp* infection plates (Supplementary Fig. S1E). After 24 hours of contact (Supplementary Fig. S1F), the seedling roots were infected by *Mp* hyphae (Fig. 1A,C). By 48 hours, hyphae penetration continued and microsclerotia could be seen forming in some Arabidopsis roots (Fig. 1E). Root tissue was flash frozen in liquid nitrogen at 24 hpc and 48 hpc for RNA-seq or at 48 hpc for qPCR analysis (Supplementary Fig. S1H). Alternatively, at four-five days post contact (dpc) microsclerotia density was determined by counting the number of microsclerotia per mm in each seedling’s primary root (Supplementary Fig. S1G). Infection plates typically contained 12–14 seedlings, and the microsclerotia per mm density was based on the average of all tested seedlings per plant line (at least three plates per line) in a biological replicate.

### Arabidopsis mutants compromised in ET signaling show enhanced root susceptibility to *Mp*

**Figure 2:**
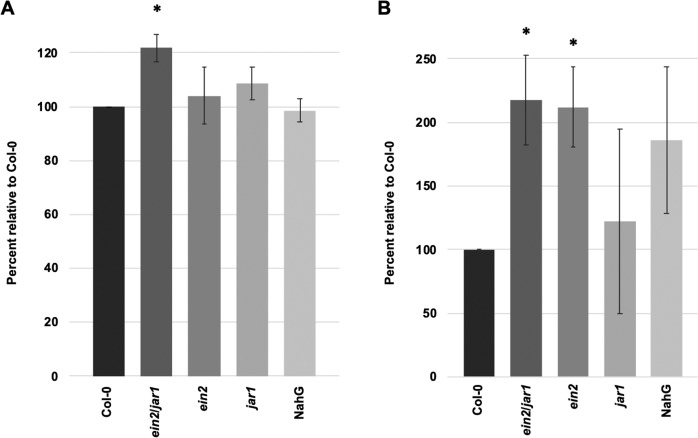
*Mp* growth quantification using an agar plate pathosystem and SCAR-qPCR identified mutant plant line(s) with increased susceptibility to *Mp*. (**A**) *Mp* microsclerotia were counted under microscopy in individual seedling primary roots, 4–5 days post-contact (dpc). The average percent microsclerotia per mm root relative to Col-0 is shown. (**B**) The relative abundance of *Mp* and Arabidopsis genomic DNA was determined by the quantification of sequence characterized amplified regions (SCAR) of *Mp* (*MpSyk*)^51^ and Arabidopsis (Shaggy-related Kinase 11, *AtSK11*)^52^ by qPCR using Arabidopsis roots 48 hpc with *Mp*. Error bars represent standard error for three biological replicates. Statistical significance relative to Col-0 determined by Student’s t-test, asterisk; *p* < 0.05.

To identify host defense mechanisms that affect levels of *Mp* susceptibility in Arabidopsis roots, we tested a set of Arabidopsis Col-0 mutants. As *Mp* is known to mainly exhibit a necrotrophic lifestyle, we tested the *jar1* and *ein2* single mutants as well as the *ein2*/*jar1* double mutant with defects in signaling processes mediated by JA or/and ET, respectively (Fig. 2A). Mutants of *JAR1* (Jasmonoyl isoleucine conjugate synthase1) are compromised in the conversion of (+)-7-iso-JA to (+)-7-iso-jasmonoyl-L-isoleucine (JA-Ile), which is one of the main bioactive forms of JA^47^. The *jar1* mutant is known to exhibit complete loss of JA signaling in Arabidopsis roots^48^. The ER-associated EIN2 (ET INsensitive 2) protein links signaling processes triggered by various ET receptors to EIN3-EIL1-type transcription factors^49^. Null mutants of *EIN2*, including the *ein2* lines used here, are completely ET insensitive^50^.

In the *ein2/jar1* double mutant, but not in the *ein2* or *jar1* single mutants, we observed significantly increased density of microsclerotia in roots (Fig. 2A). We confirmed this finding by comparing the abundance of Arabidopsis- and *Mp*-specific genomic DNA in Arabidopsis roots 48 h after *Mp* contact using quantitative PCR (qPCR) with primer pairs specifically targeting species-specific sequence characterized amplified regions (SCAR) of *Mp* (*MpSyk*) and Arabidopsis (Shaggy-related Kinase 11, *AtSK11*) DNA^51,52^ (Fig. 2B). This SCAR-qPCR assay showed relative abundance of *Mp*-specific DNA (compared to Arabidopsis-specific DNA) to be clearly elevated in *Mp*-infected roots of the *ein2/jar1* line compared to Col-0 plants indicating twice the amount of *Mp* biomass in this mutant. Contrary to our microsclerotia density assay, we also observed a significant increase of relative *Mp* DNA levels in the *ein2* single mutant compared to Col-0 (Fig. 2B). We attribute this to the fact that the SCAR-qPCR assay is more sensitive than microsclerotia density measurements and detect DNA from other *Mp* structures, such as hyphae. While neither the microsclerotia density assay (Fig. 2A), nor SCAR-qPCR (Fig. 2B) indicated significantly enhanced susceptibility in the *jar1* single mutant, this mutant exhibited a large degree of *Mp* biomass variability in the SCAR-qPCR. We also included, in both assays, the transgenic NahG line^53^ which does not accumulate the defense hormone SA, but did not observe any significant effect. However, as in the case of *jar1*, our SCAR-qPCR assay revealed that *Mp* biomass variability was unusually strong in NahG plants (Fig. 2B).

Collectively, in our agar plate-based assay system, we observed enhanced levels of *Mp* growth in mutants compromised in ET signaling, showing that this stress signaling pathway contributes to defense reactions in Arabidopsis roots against *Mp*.

### Arabidopsis mutants compromised in ET-, JA- and SA signaling show enhanced susceptibility to *Mp* in shoots

**Figure 3:**
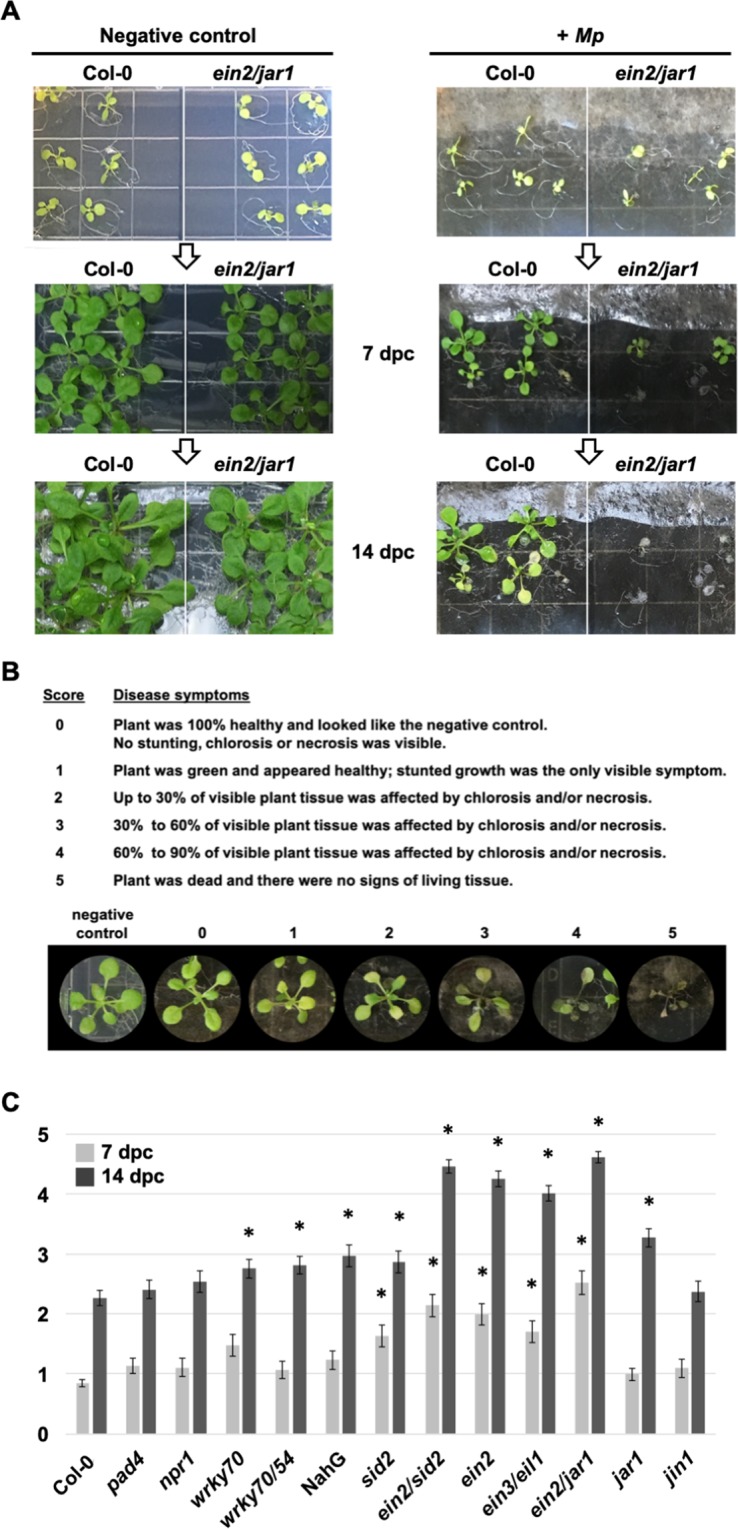
Arabidopsis shoot disease symptom severity was higher in mutants than in Col-0. (**A**) Ten day-old seedlings were placed on ½ MS agar plates (left) or *Mp* infection plates (right) and scored 7 and 14 days post-contact (dpc) for shoot disease symptom severity. Representative plates (one untreated and one infection plate) are shown. (**B**) Arabidopsis shoot disease index system with representative plant score photos. (**C**) Plants were scored at 7 dpc (grey bars) and at 14 dpc (black bars) with *Mp*. Error bars represent standard error for at least three biological replicates, n = 30. Statistical significance relative to Col-0 was determined using the Wilcoxon Rank Sum test for two independent samples, asterisk; *p* < 0.05.

In order to investigate *Mp*-induced damage to aerial plant tissues, since shoot damage is a common visual indicator of disease in many *Mp*-infected crop plants in the field^16^, we repositioned the seedlings and increased the length of time on *Mp* infection plates. Despite the still-abundant level of *Mp* on infection plates, these few changes allowed for a more gradual development of disease symptoms with visible phenotype variation in shoots (i.e., differing rates of decay) between lines (Fig. 3). Similar to the root assays, ten day old Arabidopsis seedlings were transferred to *Mp* infection plates; however, plants were allowed to grow for two weeks with plates positioned right-side-up under growth room conditions. Seedlings were photographed during the infection period and their disease severity was determined. Using a disease index scoring system of “0”, for healthy plants, through “5”, for dead plants, (Fig. 3B), we compared disease symptoms (e.g., chlorosis, necrosis, stunting) between Col-0 and mutant lines. Of the six mutants with defects in ET and/or JA-signaling that we tested, five (*ein2/sid2*, *ein2*, *ein3/eil1*, *ein2/jar1*, *jar1*) exhibited more severe disease symptoms (average disease index scores of 4.5, 4.3, 4.0, 4.6, 3.3, respectively) than Col-0 (wild type, average disease index score: 2.26) after two weeks (Fig. 3C). In some cases, the effect was already clear after 1 week. After two weeks of *Mp* infection, *ein2/jar1* had the most severe disease symptoms and many of the *ein2/jar1* (81%)*, ein2* (70%) *and jar1* (34%) plants were dying (disease index score 4.5–5; Col-0, 7%) (Fig. 3A). In addition to these mutants, which are compromised in upstream ET- and/or JA-signaling processes, we also observed clearly enhanced *Mp* susceptibility in the *ein3/eil1* double mutant (Fig. 3C), which is deficient in EIN3 and EIL1^54^. Both of these related transcription factors control almost all ET-responses as well as some responses to JA^55^. However, we did not observe any significant change of *Mp* susceptibility levels in *jin1*, a mutant deficient in the transcription factor Myc2, which mediates JA-triggered wound responses^56^.

Shoot disease symptoms were less severe in SA-deficient NahG plants than in those of the JA and ET mutants, yet were also significantly higher than Col-0 plants at the two-week time point (Fig. 3C). To confirm a potential role of SA in host defenses against *Mp* we tested additional SA signaling deficient Arabidopsis lines and observed enhanced levels of *Mp* susceptibility relative to Col-0 in the *sid2*-2, *wrky70* and *wrky70/54* mutants (Fig. 3C). The *sid2-2* mutant is deficient in isochorismate synthase 1, an enzyme catalyzing a key step in defense-induced SA biosynthesis^57^, while the tested *wrky* mutants are blocked in regulatory steps downstream from SA, with WRKY70 and WRKY54 being two closely related TFs mediating SA-responsive gene expression^58,59^. Given that SA contributes to elevated *Mp* tolerance in our experiments, the lack of significant effects of *npr1* and *pad4* plants in the shoot disease index assay was unanticipated. Yet, while NPR1 and PAD4 are both known to contribute to SA-dependent immunity, their significance for certain defense mechanisms seems variable. NPR1 is indispensable for SA-dependent systemic immunity, but its importance for local defense reactions may be limited^60^. The roles of PAD4 appear more complex, as it is also known to contribute to SA-independent processes, which may negatively interfere with efficient *Mp*-directed host immunity^61–63^.

### Profiling of *Mp*-induced transcriptome changes in Arabidopsis roots

In order to identify host transcriptome changes associated with defense responses against *Mp* in Arabidopsis roots, we performed mRNA-seq with poly-A mRNA from agar plate-grown Arabidopsis Col-0 or *ein2/jar1* roots at 24 h or 48 h after *Mp* contact or control treatment. For each of the eight different experimental conditions two independent biological replicates were performed. A comparison between levels of Arabidopsis and *Mp*-specific 18S RNAs at 48 h post contact in samples used for this analysis indicated a nearly two-fold higher abundance of *Mp* in *ein2/jar1* roots compared to roots of Col-0 (Supplementary Fig. S3). This observation further confirms that the *ein2/jar1* double mutant exhibits elevated susceptibility to *Mp* and shows that the treatments for our transcript profiling experiment were successful.

To identify gene expression changes potentially relevant for the induction of heightened resistance in Col-0 we performed the following pair-wise comparisons: (1) Col-0 24 hpc with *Mp*/Col-0 24 h untreated; (2) Col-0 48 hpc with *Mp*/Col-0 48 h untreated; (3) *ein2/jar1* 24 hpc with *Mp*/*ein2/jar1* 24 h untreated; (4) *ein2/jar1* 48 hpc with *Mp*/*ein2/jar1* 48 h untreated; (5) Col-0 48 h untreated/Col-0 24 h untreated; (6) Col-0 48 hpc with *Mp*/Col-0 24 hpc with *Mp*; (7) *ein2/jar1* 48 h untreated/*ein2/jar1* 24 h untreated; (8) *ein2/jar1* 48 hpc with *Mp*/*ein2/jar1* 24 hpc with *Mp*; (9) *ein2/jar1* 24 hpc with *Mp* / Col-0 24 hpc with *Mp*; (10) *ein2/jar1* 48 hpc with *Mp*/Col-0 48 hpc with *Mp*; (11) *ein2/jar1* 24 h untreated/Col-0 24 h untreated; (12) *ein2/jar1* 48 h untreated/Col-0 48 h untreated; (Supplementary Tables S1–S12 and Fig. S4). Transcript level differences in these comparisons with an adjusted *p* value of less than 0.05 (Table 1) were considered significant.

**Table 1:** RNA-seq twelve comparisons of significant gene expression changes.

Comparison	Total significant* DEGs	Up-regulated DEGs	Down-regulated DEGs
Col-0 24 hpc with *Mp*/Col-0 24 h untreated	335	260	75
Col-0 48 hpc with *Mp*/Col-0 48 h untreated	6,227	3,349	2,878
*ein2/jar1* 24 hpc with *Mp*/*ein2/jar1* 24 h untreated	421	393	28
*ein2/jar1* 48 hpc with *Mp*/*ein2/jar1* 48 h untreated	8,183	4,584	3,599
Col-0 48 h untreated/Col-0 24 h untreated	210	173	37
Col-0 48 hpc with *Mp*/Col-0 24 hpc with *Mp*	29	6	23
*ein2/jar1* 48 h untreated/*ein2/jar1* 24 h untreated	57	37	20
*ein2/jar1* 48 hpc with *Mp*/*ein2/jar1* 24 hpc with *Mp*	16	5	11
*ein2/jar1* 24 hpc with *Mp*/Col-0 24 hpc with *Mp*	24	5	19
*ein2/jar1* 48 hpc with *Mp*/Col-0 48 hpc with *Mp*	273	146	127
*ein2/jar1* 24 h untreated/Col-0 24 h untreated	56	10	46
*ein2/jar1* 48 h untreated/Col-0 48 h untreated	853	93	760

^*^Adjusted *p* value < 0.05.

**Figure 4:**
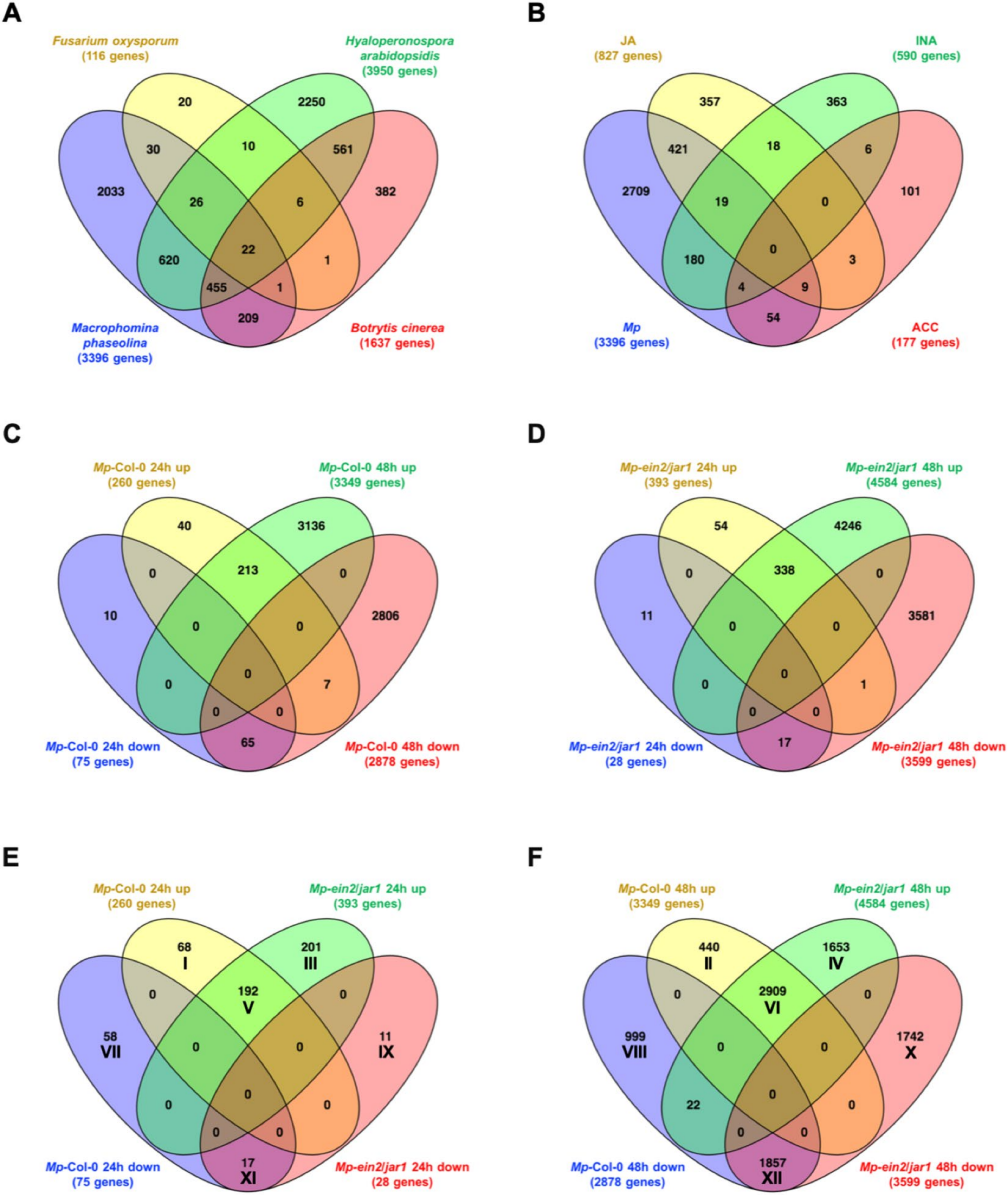
Transcript profiling comparisons reveal overlaps of transcriptome up-regulation between pathogen and hormone treatment assays with that of *Mp*-infection and between Col-0 and *ein2/jar1* at 24 or 48 hours post contact (hpc) with *Mp*. (**A**) Treatment of Col-0 seedlings with pathogens of varying lifestyles and in different plant tissues show different degrees of transcriptome up-regulation compared to *Mp*-infection. *Fusarium* *oxysporum*^64^ overlap by 68% (79/116), *Hyaloperonospora* *arabidopsidis*^66^ by 28% (1,123/3,950) and *Botrytis cinerea*^65^ by 42% (687/1637). (**B**) Hormone treatments with jasmonic acid (JA)^67^ overlap by 54% (449/827), 1-aminocyclopropane-1-carboxylic acid (ACC)^63^ overlap by 38% (67/177) and 2,6- dichloroisonicotinic acid (INA)^69^ overlap by 34% (203/590). (**C**-**F**) Venn diagram analysis illustrates differences and similarities between wild type and *ein2/jar1 Mp* infection. (**C**) Col-0 24 h untreated vs. Col-0 24 hpc with *Mp* compared to Col-0 48 h untreated vs. Col-0 48 hpc with *Mp*. (**D**) *ein2/jar1* 24 h untreated vs. *ein2/jar1* 24 hpc with *Mp* compared to *ein2/jar1* 48 h untreated vs. *ein2/jar1* 48 hpc with *Mp*. (**E**) 24 h Col-0 (from (**C**)) compared to 24 h *ein2/jar1* (from (**D**)). (**F**) 48 h Col-0 (from (**C**)) compared to 48 h *ein2/jar1* (from (**D**)).

The set of Arabidopsis Col-0 genes up-regulated in response to *Mp* infection at either 24 hpc or 48 hpc (3,396 genes) seems to a certain extent unique, as it shows only partial overlap with gene sets up-regulated by other pathogens, such as the necrotrophic fungi *Fusarium oxysporum* and *Botrytis cinerea* or the biotrophic oomycete *Hyaloperonospora arbidopsidis* (*Hpa*) (Fig. 4A). Of the three comparisons in Fig. 4A, the gene set induced by *F. oxysporum*^64^, has the largest relative overlap with the *Mp*-induced transcript profile in Col-0 roots, as 68% (79/116) of *F. oxysporum*-induced genes are also up-regulated by *Mp*. *F. oxysporum* and *Mp* are both necrotrophic, soil-borne fungal pathogens that often infect plants through their roots. The overlap of genes up-regulated in aerial parts of Arabidopsis by the nectrotrophic fungus *B. cinerea*^65^ with *Mp*-induced genes is less extensive at 42% (687/1,637), which, however, may partially reflect the fact that different types of host tissues were used. A relatively low percentage (28%; 1,123/3,950) of Arabidopsis genes induced by *Hpa*^66^ are also up-regulated by *Mp*. This is not surprising given that *Hpa* is a biotrophic oomycete that infects aerial portions of the plants and exhibits a very different lifestyle from *Mp*. Again, these differences also likely reflect that different host tissue types were profiled. Despite the fact that the relative overlap of these two gene sets is small, a large absolute number of genes (1,123) are jointly up-regulated in both types of infections indicating that some host defense responses may be inducible by both of these dissimilar types of pathogens.

Similarly, a comparison of the same set of *Mp*-induced genes to those up-regulated in hormone-treated Arabidopsis revealed commonalities of *Mp*-induced responses to those activated by other defense stimuli (Fig. 4B). Our comparison shows 54% (449/827) of a set of Arabidopsis genes induced by JA^67^ overlap with our *Mp* inducible gene set. Of 177 genes induced in Arabidopsis by the ET precursor 1-aminocyclopropane-1-carboxylic acid (ACC)^68^, 67, or ~38%, overlap with the set of *Mp* –up-regulated genes (Fig. 4B), while, of 590 Arabidopsis genes up-regulated by the SA analog INA^69^, 203 (34%) of which overlap with *Mp* inducible genes. These observations further confirm a likely role of ET and JA as critical regulators of immune responses against *Mp*, but also suggest that parts of the canonical SA-dependent defense system also may be activated in response to *Mp* infections.

### Promoter-motif enrichment analysis with *Mp*-responsive gene sets

Enrichment of sequence motifs representing certain transcription factor (TF) binding sites in promoter regions of *Mp*-responsive genes further supports a combined role of JA, ET and SA in controlling transcriptome changes induced by this pathogen. TFs typically bind specifically to short DNA stretches of defined sequence, but also exhibit some ambiguity binding to a range of derivatives of a certain type of DNA motif. While any sequence motif is randomly distributed in genomic DNA, statistical enrichment of a given motif in promoter regions of genes that are co-expressed under certain conditions, can indicate a role for this motif (and cognate binding TFs) in directing this expression pattern^70^.

JA-responses are often mediated by Myc2 and related bHLH TFs, which have a strong binding preference for the well-characterized G box (CACGTG)^56,71^. Transcriptional up-regulation in response to ET and JA is mediated by the related Arabidopsis TFs EIN3 and EIL1^54^. EIN3 (and likely also EIL1) can bind to DNA sequences containing the pentameric motif TACAT^72^. In response to ET and/or JA, EIN3/EIL1-related TFs can activate expression of members of the large ERF TF family which bind to a variety of binding sites related to the GCC box (AGCCGCC)^73^. WRKY TFs, which bind to various sequences related to the W box (TTGACC/T)^58,59^, are associated with SA-driven transcriptome changes, but can also be involved in other types of immune responsive gene expression control, such as responses mediated by PTI-associated MAP-kinase cascades. More strictly associated with SA-signaling seem to be TGA-bZIP TFs and TBF1^74,75^. While the former type of TFs are known to bind to TGA boxes (TGACG), TBF1 (and likely additional so far unknown factors) binds to TC-rich sequences, such as the TL1 element (TTCTTCTTC)^75,76^ or the related *LURP*^*A*^ element (TCTTCT)^69^.

Our set of RNA-seq data allowed us to discriminate three temporal patterns of *Mp*-induced transcript accumulation in Col-0 plants: (1) “early/transiently up” (only up-regulated by *Mp* at the 24 h time point), (2) “early/sustained up” (up-regulated at both 24 h and 48 h after *Mp* contact) and (3) “late up” (only up-regulated at 48 h after *Mp* contact). We observed strong enrichment of hexameric motifs related to all tested promoter motifs in our sets of *Mp*-response genes, except for the GCC box (Table 2). While in the promoter regions of “early/transiently” up-regulated genes only a G-box related motif seems conserved, the two remaining sets, “early/sustained up” and “late up”, feature a variety of conserved motifs related to the G-box, EIN3/EIL1 binding sites, W-box, TGA box and TC-rich motifs. Thus, the up-regulation *Mp*-responsive genes may indeed be mediated by the combined activity of known ET/JA-associated TFs (Myc2-like, EIN3/EIL1-like) as well as SA-associated (WRKY, TGA-bZIP and TC-rich motif binding proteins) TFs. These observations are consistent with the enhanced *Mp* susceptibility phenotype we observed in *ein3/eil1* and *wrky70/54* mutants and further support that host immune responses of Arabidopsis against *Mp* are mediated by the combined activity of ET/JA signaling and SA signaling pathways.

**Table 2:** Enrichment of hexameric sequence motifs representing defense-associated transcription factor (TF) binding sites^1,2^ in promoter regions of genes responsive to *Mp* in Col-0 plants.

Gene set	Conserved motif^b^ (Segments matching known motif consensus are underlined.)	Motif type/general motif consensus^a^	*p* value/comments
**early/transiently up** (47 genes up-regulated in Col-0 by *Mp* ONLY at 24 hpc AND NOT at 48 hpc)	**ACGTGG**	G box-like (CACGTG)	*p* = 4.00e-04 (top hit, 19 matches)
**early sustained up** (213 genes up-regulated in Col-0 by *Mp* at both 24 hpc AND 48 hpc)	**TTGACT**	W box (TTGACC/T)	*p* = 1.10e-07 (top hit, 143 matches)
	**ACGTTG**	G box-like (CACGTG)	*p* = 9.28e-07 (78 matches)
	**ATTGAC**	W box-like (TTGACC/T)	*p* = 1.99e-06 (123 matches)
	**ATTGAC**	TGA box-like (TGACG)	
	**CTTGAC**	W box-like (TTGACC/T)	*p* = 6.99e-06 (93 matches)
	**CTTGAC**	TGA box-like (TGACG)	
	**CTTCTC**	TL1/LURP^A^, TC-rich motifs TCTTCT	*p* = 9.22e-06 (87 matches)
**only late up**^c^ (521 genes up-regulated in Col-0 by *Mp* ONLY at 48 hpc AND NOT at 24 hpc)	**ACGTGT**	G box-like (CACGTG)	*p* = 1.02e-12 (top hit, 201 matches)
	**CACGTA**	G box-like (CACGTG)	*p* = 2.91e-07 (142 matches)
	**TAACAT**	EIN3/EIL1-binding site like (ATCAT)	*p* = 5.27e-07 (362 matches)
	**CACGTT**	G box-like (CACGTG)	*p* = 8.19e-07 (162 matches)
	**ACGTGG**	G box-like (CACGTG)	*p* = 2.79e-05 (133 matches)
	**ACGTGA**	G box-like (CACGTG)	*p* = 3.72e-05 (155 matches)
	**CTTCTC**	TL1/LURP^A^, TC-rich motifs TCTTCT	*p* = 8.39e-05 (239 matches)
	**TCTTCA**	TL1/LURP^A^, TC-rich motifs TCTTCT	*p* = 2.30e-04 (296 matches)
	**CTTCTG**	TL1/LURP^A^, TC-rich motifs TCTTCT	*p* = 3.23e-04 (149 matches)
	**AAACAT**	EIN3/EIL1-binding site like (ATCAT)	*p* = 6.90e-04 (428 matches)
	**AACATT**	EIN3/EIL1-binding site like (ATCAT)	*p* = 6.98e-04 (402 matches)
	**CTCTTC**	TL1/LURP^A^, TC-rich motifs TCTTCT	*p* = 7.76e-04 (230 matches)
	**GTTGAC**	W box-like (TTGACC/T)	*p* = 8.43e-04 (193 matches)
	**GTTGAC**	TGA box-like (TGACG)	
	**TGACGT**	TGA box (TGACG)	*p* = 9.89e-04 (132 matches)
	**CACGTG**	G box (CACGTG)	*p* = 8.37e-06 (103 matches) Slightly below the arbitrary threshold for the number of matches.

^a^Known sequence motifs considered in this analysis (listed with their cognate TF family in bold):

CACGTG (G-box***, Myc2-like***),

ATCAT (***EIN3/EIL1-like***)

G/ACCGCC (GCC box, ***ERF***)

TTGACC/T (W-box, ***WRKY***)

TGACG (TGA-box, ***TGA-bZIP***)

TCTTCT (TL1/LURP^A^, TC-rich motifs, ***TBF1, others?***).

^b^Conserved hexameric motifs were identified by TAIR Motiffinder (https://www.arabidopsis.org/tools/bulk/motiffinder/index.jsp), which identifies conserved 6mers in 1000 bb upstream sequences (only motifs with *p* values equal or less than 1e-03, which are present in 1,000 bp upstream sequences of at least 1/4 of all genes in each set are listed).

^c^Only genes showing at least a 4-fold up-regulation by *Mp* were considered, as the full set of 3,136 “only late up” genes did not lead to the identification of conserved motifs satisfying the criteria above (*p* values equal or less than 1e-03; present in 1,000 bp upstream sequences of at least 1/4 of all genes).

Despite the fact that G box-related motifs are strongly enriched in the examined sets of *Mp*-induced Arabidopsis genes, we had not observed altered *Mp*-susceptibility of the Myc2-deficient *jin1* mutant. This may reflect the complex roles of Myc2 in JA/ET-signaling, as it can suppress certain groups of genes, while activate others^56^. Lack of an *Mp* defense-related phenotype in *jin1* may also be due to possible functional redundancy between this TF and other related Myc family members^77^.

### Functional categories of *Mp*-responsive Arabidopsis genes

We further categorized all Arabidopsis genes differentially expressed after *Mp* based on the timing and dependency of their transcriptional changes on *EIN2/JAR1* into 12 groups (labeled by roman numerals I - XII in Fig. 4E,F, Supplementary Tables S13–S25) and performed a GO term enrichment analysis. Differential gene expression changes detected by our RNA-seq analysis may reflect inducible host defense responses to combat *Mp* and active host responses to counter *Mp*-inflicted disease symptoms and damage. Furthermore, such gene expression changes may also be the consequence of *Mp* effector activity and serve pathogen virulence rather than host immunity and damage protection. Several general themes stand out. Consistent with our observation that known defense responses mediated by JA, ET and SA contribute to host resistance against *Mp*, groups of *Mp*-up-regulated genes are enriched for GO terms associated with responses to defense, stress, JA, ET, SA, various pathogens and, also, wounding (a known trigger of JA signaling). Similarly, up-regulated genes associated with the strongly enriched GO term “camalexin biosynthesis” are likely serving direct host immune responses, by promoting the synthesis of this indole-related phytoalexine. Host genes up-regulated to counter *Mp*-inflicted damage may be associated with GO terms representing protein re-folding, senescence, and responses to starvation, heat or toxic substances; all of which are particularly strongly enriched in genes up-regulated by *Mp* infection in the more susceptible *ein2/jar1* line (Supplementary Table S25). Numerous Arabidopsis genes associated with GO terms related to plant cell wall metabolism are down-regulated by *Mp* infection (Supplementary Table S25). These and other gene sets related to plant growth, development, metabolism or gene expression may be actively targeted by *Mp* effectors to weaken the host and to counteract host defense responses.

## Discussion

Using agar plate-based assays for interactions between Arabidopsis and *Mp*, we found the ET/JA signaling deficient *ein2/jar1* double mutant to exhibit robustly enhanced susceptibility to this pathogen compared to its wild type Col-0 parental background. Three different quantitative/semi-quantitative assays (microsclerotia density, SCAR-qPCR and shoot disease index), provided highly consistent results uncovering enhanced fungal growth and host damage by *Mp* in this double mutant. Overall, our shoot disease index-based assay appeared to be of higher sensitivity than the root-based assays and allowed for higher throughput. Therefore, we used this assay to analyze a wider set of mutants. Using the set of assays, we also found *Mp* susceptibility to be enhanced in the *ein2* and *jar1* single mutants, which are deficient in responses to ET or JA, respectively. These results, and observations we made with additional ET- or JA-mutants, implicate ET and JA signaling as important for protection against *Mp* in Arabidopsis. Together, the assays can uncover multiple plant responses throughout the *Mp* infection process. ET-related responses are clearly evident in roots at 24 h (RNA-seq data), at 48 h (RNA-seq data and SCAR-qPCR Fig. 2B) and at 4–5 dpc (as part of the *ein2/jar1* microsclerotia density increase) as well as in shoots at 7 and 14 dpc. JA-related responses also contribute to root susceptibility, for while there was more *Mp* tissue present in the *ein2* plants than in the *jar1* plants (SCAR-qPCR Fig. 2B), microsclerotia density only increased when both signaling pathways were compromised (see *ein2/jar1* Fig. 2A), suggesting a possible relationship between JA signaling and microsclerotia formation that merits future investigation. In the shoot, *ein2* was significantly different from *ein2/jar1* at 14 dpc, though not at 7 dpc (data not shown), pointing to more of a role for JA during the later infection period.

ET- and JA-dependent signaling processes have also been implicated in defense against *Mp* in *Medicago truncatula* and *Sesamum* *indicum*^22,78^. While *Mp* induced enhanced transcript levels of known JA and ET response genes in sesame plants^22^, treatment of *M. truncatula* with methyl-JA and/or ET enhanced host survival after *Mp* infections^78^. Consistent with a role for ET- and JA-signaling in *Mp* immunity, we also observed up-regulation of numerous Arabidopsis genes related to these processes in *Mp*-infected Col-0 plants. These included genes of various ERF (Ethylene Response Factor) transcription factors, JA-responsive JAZ transcriptional regulators as well as ET or JA biosynthetic enzymes. We further found promoter motifs related to known JA- and ET-response elements to be substantially enriched in *Mp*-responsive Arabidopsis genes. Given that *Mp* is considered to exhibit a mostly necrotrophic lifestyle, it is not surprising that Arabidopsis activated ET/JA-dependent immune responses upon infection with this pathogen. Both of these stress hormones are known to control plant immune responses against necrotrophic pathogens^45,46^. Our results served as critical “proof-of-concept” demonstrating that the new model pathosystem and defense assays we developed allow for the discovery of Arabidopsis processes suppressing *Mp* infections. Furthermore, our results provided an opportunity to use an Arabidopsis line with elevated levels of susceptibility for a comparative transcriptomics study to identify gene expression changes associated with host defense reactions against *Mp*.

Surprisingly, the *Mp*-responsive Arabidopsis transcriptome exhibited substantial overlap with gene sets known to be inducible by SA. Consistent with this, we also observed strong enrichment of promoter motifs related to binding sites of SA-associated TFs in *Mp*-responsive gene sets. Our *Mp* defense assays further confirmed a role of SA signaling in host immunity against this pathogen. Given the predominantly necrotrophic life-style of *Mp*, this was somewhat unexpected, as SA is known to control immune responses against biotrophic pathogens^45,79^. However, Chowdhury and co-workers^22^ recently proposed that in interactions with sesame, *Mp* employs a short biotrophic attack strategy prior to switching to a primarily necrotrophic phase. Based on *Mp* marker gene expression and morphological features of *Mp* hyphae between 24 h and 38 h after infection of sesame, *Mp* seems to transition from a biotrophic phase to a necrotrophic phase. This “biotrophy-necrotrophy switch” (BNS) is accompanied by a change of physiological, biochemical and transcriptional responses of the host. Most importantly, BNS is associated with a transition from typical SA- to ET/JA-response gene expression in sesame. Our results are consistent with the observations and conclusions made by Chowdhury and coworkers.

When interpreting the timing of transcriptional changes in pathogen-infected plants, it is important to consider that infection events do not occur synchronously in all cells/tissue areas of the host. At any time point, only the “average” of multiple asynchronized events can be monitored. Thus, the apparent co-occurrence of SA and ET/JA-responsive gene expression at the two time points (24 hpc and 48 hpc) studied here may, in fact, reflect two successive defense gene expression states in the host. While each individual Arabidopsis root cell may be engaged in either pre-BNS or post-BNS response activities, the tested tissue as a whole may represent a mixture of response states. Chowdhury and co-workers made similar observations. High resolution time course studies, possibly monitoring gene expression events in individual Arabidopsis root cells, may have to be employed in the future to determine if an early pre-BNS phase in *Mp* is countered by SA-mediated host defenses and if later post-BNS growth of *Mp* induces ET/JA-dependent immunity in Arabidopsis roots.

Crop diseases caused by *Mp* are typically associated with drought and heat stress^16^. Thus, the induction of drought- or heat-tolerance mediating plant stress responses may be linked to immune responses against *Mp*. We indeed observed *Mp*-induced up-regulation of numerous Arabidopsis genes associated with drought and heat tolerance, such as genes encoding several DREB (Drought-Response Element Binding Protein)-type transcription factors, drought tolerance associated LEA (Late Embryogenesis Abundant) proteins, as well as various heat shock transcription factors and heat shock proteins, in the RNA-seq data. Plant tolerance to drought stress is partially controlled by the phytohormone abscisic acid (ABA). Among genes up-regulated by *Mp* in Col-0 were genes encoding the ABA-responsive basic helix loop helix transcription factor BHLH17, the ABA-responsive protein ABR and AFP4 (a negative regulator of ABA-responses). Future reverse genetic studies may be performed to test if ABA-signaling or other known abiotic stress response mechanisms affect the outcome of *Mp*-Arabidopsis interactions. In any case, our results point toward a complex interplay of multiple plant hormone-controlled signaling processes in immunity against *Mp*.

Having established the Arabidopsis-*Mp* interaction as a lab model will allow for faster progress in uncovering plant defenses against this detrimental pathogen. In this study, advantages of the Arabidopsis system became obvious. An abundance of well-characterized mutants with defined defects in signaling pathways enabled targeting of certain candidate processes (e.g. ET- and JA-signaling) by reverse genetic analyses. The existence of a well-annotated Arabidopsis genome sequence allowed for extraction of detailed information from the RNA-seq based transcriptomics experiments. Furthermore, a wealth of existing RNA-seq and microarray data was available for a wide variety of biological conditions in Arabidopsis for comparison to our results.

Next steps in the use of the new model phyto-pathosystem will include systematic testing of candidate genes identified by the RNA-seq study for their contribution to *Mp* defense responses using existing sequence-indexed T-DNA mutant collections^80–82^, which collectively cover almost 100% of Arabidopsis genes. The same reverse genetics resources can be used to test conserved QTL candidate loci potentially protecting crop species against *Mp*. Comprehensive collections of natural Arabidopsis accessions (ecotypes) can be tested against a panel of different *Mp* isolates to identify race-specific interactions with differential outcomes. Existing SNP resources for many Arabidopsis accessions or recombinant inbred lines^83^ can be used to map loci responsible for the observed differential effects. Finally, Arabidopsis has proven to be an excellent system for high throughput applications, such as forward genetic screens of large populations of randomly mutagenized individuals^84^ or chemical screens^69,85^. Mutations or chemicals, respectively, affecting the outcome of plant-*Mp* interactions, can be identified this way. We have begun some of these approaches.

Although artificial, the agar plate-based Arabidopsis root assays provided conditions for *Mp* to engage in its natural mode of host infection, as microsclerotia formed hyphae, which penetrated dermal root tissues resulting in colonization (as seen in soybean^38^, sesame^19^, and *M. truncatula*^78^*)* and the formation of new microsclerotia within the host. Thus, molecular host responses identified in these assays are likely to reflect authentic processes occurring during plant-*Mp* interactions under natural conditions. Studying the plant-pathogen interaction on agar plates removed interference from unknown structures, compounds or organisms present in autoclaved soil that could provide additional variables affecting plant responses or pathogen behavior. The *Mp*/Arabidopsis experimental system established here should allow researchers to make great progress regarding the identification of key genes affecting the outcome of plant-*Mp* interactions. Knowledge of such genes in Arabidopsis and their orthologs in crop species will facilitate the design of new molecular markers for precise marker-based breeding approaches in economically important plants. New synthetic elicitors may also be identified that specifically activate plant immune responses active against *Mp* and which could serve as leads for the development of new pesticide alternatives. Beyond benefiting agriculture directly, discoveries made using our experimental pathosystem may allow for the gain of deeper insight into immune responses against *Mp*. The potential interplay of various hormone-pathways, which is involved in controlling immunity against *Mp*, is a particularly appealing subject that can be approached using our agar-plate-based *Mp*/Arabidopsis infection assays.

## Methods

### Plant and pathogen growth conditions

The Arabidopsis double mutant line *ein2*-1/*jar1*-1 has been described^86^. All mutants are in the Arabidopsis ecotype Columbia (Col-0) background: *ein2*-1^87^, *ein3*-1/*eil1*-1^88^, *jar1*-1^89^, *jin1*^90^, *pad4*-1^91,92^, *sid2*-2^93^, *ein2*-1/*sid2*-2^94^, *npr1*-3^95,96^, NahG^53^, *wrky70*-1^80,97^ and *wrky70/*54^98^. Growth room conditions were under fluorescent lights (16 h of light/8 h of dark, 23 °C, 100 μE m^−2^ s^−1^). Seeds were surface-sterilized in 70% ethanol and a 0.02% Triton X, 20% bleach solution, for three and ten minutes, respectively, followed by sterile water rinses. Seeds were then plated on solid media containing ½ MS (Murashige and Skoog), 0.05% MES, 0.25% sucrose and 0.87% agar; pH was 5.7 prior to the addition of agar. All ½ MS-agar plates contained approximately 25 ml of media. Plated seeds were stratified for two days (or six days for accessions other than Col-0) at 4 °C before plates were positioned vertically under growth room conditions for 10 days. *Mp*-infected roots were placed in acid fuchsin stain^99^ 5 dpc for 18 hours followed by destaining in water.

*Mp* (originally isolated from UCR Ag Operations Field 11 by members of Philip Roberts’ laboratory^16^) was propagated by adding a plug to potato dextrose agar (BD Difco^TM^, http://us.vwr.com) -containing plates (10 ml per plate), incubating at 34 °C for 10 days and then allowed to dry at RT for at least four weeks.

### *Mp* infection assays

*Mp* infection plates were created by adding *Mp* inoculum (approximately 2000 microsclerotia in 2–3 ml media for microsclerotia counting and RNA-seq roots and twice the amount for the shoot disease index assay) to plates, placing plates in a dark incubator at 34 °C for three days and then transferring the plates to growth room conditions for an additional three days. The media used to pour plates and create inoculum contained ½ MS (Murashige and Skoog), 0.05% MES, 0.25% sucrose and 0.87% agar; pH was 5.7 prior to the addition of agar. Dried *Mp* propagation plate contents were ground in 10–20 ml sterile water with mortar and pestle, added to media and counted in 100μl drops on slides until the desired concentration was reached.

For the microsclerotia density assay, 10 day-old seedlings were gently transferred to *Mp* infection plates, with 12–14 plants per plate, and these plates were placed vertically in growth room conditions for four to five days. To arrest *Mp* development, 20–30 ml 75% EtOH was added to each plate. Microsclerotia were counted under bright field microscopy and root lengths were measured using ImageJ^100^. Magnified images were taken using a Leica DM/LB2 (Leica, Wetzlar, Germany) microscope equipped with an RT colour SPOT camera. At least three biological replicates were conducted with a minimum of three plates per line. Statistical significance relative to Col-0 was determined by Student’s t-test, *p* < 0.05.

For the *Mp*-Arabidopsis shoot disease index, 10 day-old seedlings were gently transferred to *Mp* infection plates, with two lines per plate, 10 plants per line (n = 30), and these plates were placed horizontally in growth room conditions for two weeks. Images were taken of whole plates throughout the infection period to monitor progress of disease symptoms in plants. Individual plants were assessed according to a disease index scoring system of “0”, for healthy plants, through “5”, for dead plants (Fig. 3B). At least three biological replicates were conducted and significance relative to Col-0 was determined using the Wilcoxon Rank Sum test for two independent samples by Mathcracker on https://mathcracker.com/wilcoxon-rank-sum#results.

### Transcriptome profiling by mRNA-seq

For RNA-seq, 10 day-old seedlings were transferred to agar plates without *Mp* or to *Mp* infection plates, with 12–14 plants per plate, and these plates were placed vertically in growth room conditions for 24 h or 48 h. Plant roots were separated from shoot tissue using a blade and flash frozen in liquid nitrogen at 24 h or 48 h post transfer. Total RNA was isolated from roots using TRIzol (Invitrogen^TM^, http://www.thermofisher.com). RNA was processed (74204 QIAGEN, http://www.qiagen.com, and AM1907 Invitrogen^TM^, http://www.thermofisher.com) and libraries were prepared with the NEBNext Ultra Directional RNA Library Prep Kit for Illumina by following the manufacturer’s instruction (E7490S, E7335S, E7420S, New England Biolabs, http://www.neb.com). Root tissues were separately analyzed for each line (Col-0 or *ein2*-1/*jar1*-1), treatment (+/− *Mp*) and time point (24 h or 48 h). Two independent biological replicates, with three technical replicates for each experimental condition, were performed.

Libraries were pooled and sequenced on an Illumina NextSeq. 500 (Illumina, San Diego, CA, USA) platform at the UCR Genomics Core Facility. Reads that passed Illumina’s quality control filters were further processed. The quality of sequencing reads was assessed using FastQC v 0.11.5 (http://www.bioinformatics.babraham.ac.uk/projects/fastqc/). Unique reads were mapped to the Arabidopsis genome (TAIR10) using STAR v 2.5.3a^101^ with default settings and a known splice site file, built from Araport annotation file v11. Reads in gene regions were counted using featureCounts^102^. The expression fold‐change of each gene was calculated using the R package DESeq. 2 v1.14.1^103^ with the threshold for differentially expressed genes set to *p* value < 0.05.

The MA-plots were generated with the function *plotMA* in DESeq. 2 and illustrate log-fold change (M-values, i.e. the log of the ratio of level counts for each gene between two samples) against the log-average (A-values, i.e. the average level counts for each gene across the two samples). They showed the log_2_ fold changes attributable to a given variable over the mean of normalized counts for the compared samples. Differentially expressed gene points were red if the adjusted *p* value was less than 0.01. Genes with similar expression levels in two samples appeared around the horizontal line y = 0. Data comparison AGIs can be found in Supplementary Tables S5–S12.

Comparisons between experimental gene sets and sets of genes responding to other stimuli were done using Venny 2.1, (http://bioinfogp.cnb.csic.es/tools/venny/index.html).

Supplementary Table S25 groups I-XII GO terminology was compiled at http://go.pantherdb.org/ with the annotation version GO Ontology database, released 2018-04-04, analysis type: PANTHER Overrepresentation Test (released 20171205), test type GO biological process complete with Fisher’s Exact FDR multiple test correction displaying only results with a false discovery rate < 0.05. Supplementary Table S25 groups I-XII AGIs are listed in Supplementary Tables S13–S24.

### Real-time PCR quantification of *Mp* biomass

Arabidopsis roots were harvested 48 hpc with *Mp* for genomic DNA extraction. Genomic DNA was extracted using a modified cetyltrimethylammonium bromide (CTAB) method^104^, 1% PVP (chloroform/isoamylalcohol) was added in the 2% CTAB extraction buffer prior to use. After RNase A digestion (19101 QIAGEN http://www.qiagen.com), 20 ng of genomic DNA were used for qPCR amplification using the CFX Connect detection system (Bio-Rad) with iQ SYBR Green Supermix (Bio-Rad). Two pairs of species-specific primers were used; for *Mp* DNA amplification: MpSyk-F 5′- ATCCTGTCGGACTGTTCCAG-3′ and MpSyk-R 5′- CTGTCGGAGAAACCGAAGAC-3′; for Arabidopsis DNA amplification: AtSK11-F 5′-CTTATCGGATTTCTCTATGTTTGGC-3′ and AtSK11-R 5′- GAGCTCCTGTTTATTTAACTTGTACATACC-3′. Melt curve analysis was performed following 40 cycles of amplification with the annealing temperature at 60 °C. The ratios of *Mp* and Arabidopsis genomic DNA were calculated by the standard curve method. Serial dilutions of *Mp* and Arabidopsis genomic DNAs were used for standard curve generation (Supplementary Fig. S2). The relative amounts of *Mp* and Arabidopsis genomic DNA were calculated by normalizing *Mp MpSyk*^51^ to Arabidopsis *AtSK11* measured by qPCR as described in Gachon & Saindrenan, 2004^52^.

### Accession numbers

The Gene Expression Omnibus (GEO) accession number for RNA-seq data reported in this study is GSE127574.

## Supplementary information

Supplementary Information

Supplementary Tables 1–12

Supplementary Tables 13–24
