# Supplementary material for: A novel Arabidopsis pathosystem reveals cooperation of multiple hormonal response-pathways in host resistance against the global crop destroyer Macrophomina phaseolina
Source: Sci Rep. 2019 Dec 27;9:20083. doi: 10.1038/s41598-019-56401-2 (PMC6934584; doi:10.1038/s41598-019-56401-2)
Supplement: Supplementary file 1 — Supplementary Information [file 41598_2019_56401_MOESM1_ESM.docx]

**Supplementary Information**

Article title: A novel Arabidopsis pathosystem reveals cooperation of multiple hormonal response-pathways in host resistance against the global crop destroyer *Macrophomina phaseolina*

Authors: Mercedes M. Schroeder, Yan Lai, Miwa Shirai, Natalie Alsalek, Tokuji Tsuchiya, Philip Roberts, Thomas Eulgem

The following Supplementary Information is available for this article:

**Supplementary Tables**

(Supplementary Tables S1 – S12 are grouped in one Excel file.)

**Table S1.** Comparison 1: Col-0 24 hpc with *Mp* / Col-0 24 h.

**Table S2.** Comparison 2: Col-0 48 hpc with *Mp* / Col-0 48 h.

**Table S3.** Comparison 3: *ein2/jar1* 24 hpc with *Mp* / *ein2/jar1* 24 h.

**Table S4.** Comparison 3: *ein2/jar1* 48 hpc with *Mp* / *ein2/jar1* 48 h.

**Table S5.** Comparison 5: Col-0 48 h / Col-0 24 h.

**Table S6.** Comparison 6: Col-0 48 hpc with *Mp* / Col-0 24 hpc with *Mp*.

**Table S7.** Comparison 7: *ein2/jar1* 48 h / *ein2/jar1* 24 h.

**Table S8.** Comparison 8: *ein2/jar1* 48 hpc with *Mp* / *ein2/jar1* 24 hpc with *Mp*.

**Table S9.** Comparison 9: *ein2/jar1* 24 hpc with *Mp* / Col-0 24 hpc with *Mp*.

**Table S10.** Comparison 10: *ein2/jar1* 48 hpc with *Mp* / Col-0 48 hpc with *Mp*.

**Table S11.** Comparison 11: *ein2/jar1* 24 h / Col-0 24 h.

**Table S12.** Comparison 12: *ein2/jar1* 48 h / Col-0 48 h.

(Supplementary Tables S13 – S24 are grouped in one Excel file.)

**Table S13.** Group I: genes up-regulated at 24 h in Col-0.

**Table S14.** Group II: genes up-regulated at 48 h in Col-0.

**Table S15.** Group III: genes up-regulated at 24 h in *ein2/jar1*.

**Table S16.** Group IV: genes up-regulated at 48 h in *ein2/jar1*.

**Table S17.** Group V: genes up-regulated at 24 h in both Col-0 and *ein2/jar1*.

**Table S18.** Group VI: genes up-regulated at 48 h in both Col-0 and *ein2/jar1*.

**Table S19.** Group VII: genes down-regulated at 24 h in Col-0.

**Table S20.** Group VIII: genes down-regulated at 48 h in Col-0.

**Table S21.** Group IX: genes down-regulated at 24 h in *ein2/jar1*.

**Table S22.** Group X: genes down-regulated at 48 h in *ein2/jar1*.

**Table S23.** Group XI: genes down-regulated at 24 h in both Col-0 and *ein2/jar1*.

**Table S24.** Group XII: genes down-regulated at 48 h in both Col-0 and *ein2/jar1*.

**Supplementary Table S25.** GO terms enriched in groups I - XII.

| **Group** | **Description** | **No. genes** | **GO terms of enriched functional classes** | **Fold Enrichment** | ****Number of related categories also significantly enriched** |
| --- | --- | --- | --- | --- | --- |
| I | 24 h, up only in Col-0 | 68 | response to wounding | 13.9 | 0 |
|  |  |  | response to jasmonic acid | 13.84 | 0 |
|  |  |  | defense response | 3.85 | 0 |
|  |  |  | response to stress | 3.21 | 0 |
|  |  |  | response to stimulus | 2.05 | 0 |
|  |  |  |  |  |  |
| II | 48 h, up only in Col-0 | 440 | triterpenoid biosynthetic process | 22.58 | 1 |
|  |  |  | negative regulation of ethylene-activated signaling pathway | 19.92 | 4 |
|  |  |  | tetrahydrofolate biosynthetic process | 15.94 | 0 |
|  |  |  | cellular response to starvation | 5.02 | 6 |
|  |  |  | ethylene-activated signaling pathway | 4.52 | 8 |
|  |  |  | transmembrane transport | 2.42 | 0 |
|  |  |  |  |  |  |
| III | 24 h, up only in *ein2/jar1* | 201 | cellular response to sucrose starvation | >100 | 0 |
|  |  |  | tryptophan catabolic process | 52.62 | 19 |
|  |  |  | camalexin biosynthetic process | 38.27 | 7 |
|  |  |  | jasmonic acid and ethylene-dependent systemic resistance | 28.06 | 5 |
|  |  |  | response to absence of light | 16.70 | 0 |
|  |  |  | response to hydrogen peroxide | 14.44 | 7 |
|  |  |  | response to high light intensity | 8.16 | 1 |
|  |  |  | response to heat | 5.87 | 1 |
|  |  |  | defense response to bacterium | 4.53 | 9 |
|  |  |  | response to ethylene | 4.17 | 3 |
|  |  |  | response to acid chemical | 2.99 | 2 |
|  |  |  |  |  |  |
| IV | 48 h, up only in *ein2/jar1* | 1,653 | defense response by callose deposition in cell wall | 7.67 | 2 |
|  |  |  | response to chitin | 4.41 | 3 |
|  |  |  | plant-type hypersensitive response | 4.14 | 3 |
|  |  |  | autophagy | 4.02 | 1 |
|  |  |  | response to oomycetes | 3.97 | 0 |
|  |  |  | negative regulation of defense response | 3.64 | 1 |
|  |  |  | regulation of response to biotic stimulus | 3.29 | 0 |
|  |  |  | toxin metabolic process | 3.21 | 0 |
|  |  |  | positive regulation of innate immune response | 3.16 | 9 |
|  |  |  | regulation of response to external stimulus | 3.09 | 1 |
|  |  |  | regulation of programmed cell death | 3.02 | 1 |
|  |  |  | cell surface receptor signaling pathway | 2.81 | 0 |
|  |  |  | defense response to bacterium | 2.62 | 1 |
|  |  |  | defense response, incompatible interaction | 2.61 | 4 |
|  |  |  | protein autophosphorylation | 2.48 | 2 |
|  |  |  | response to wounding | 2.30 | 0 |
|  |  |  | protein ubiquitination | 2.07 | 2 |
|  |  |  |  |  |  |
| V | 24 h, up in both Col-0 and *ein2/jar1* | 192 | protein refolding | 20.39 | 0 |
|  |  |  | cellular response to unfolded protein | 17.92 | 3 |
|  |  |  | response to hydrogen peroxide | 15.22 | 5 |
|  |  |  | response to virus | 11.09 | 0 |
|  |  |  | cellular response to heat | 10.87 | 2 |
|  |  |  | glutathione metabolic process | 10.87 | 0 |
|  |  |  | toxin metabolic process | 10.41 | 0 |
|  |  |  | response to toxic substance | 10.33 | 11 |
|  |  |  | response to high light intensity | 10.32 | 0 |
|  |  |  | antibiotic catabolic process | 9.15 | 0 |
|  |  |  | cofactor catabolic process | 7.46 | 1 |
|  |  |  | aging | 6.76 | 0 |
|  |  |  | drug catabolic process | 5.30 | 0 |
|  |  |  | response to cadmium ion | 4.36 | 2 |
|  |  |  | defense response to bacterium | 4.34 | 8 |
|  |  |  | response to acid chemical | 2.52 | 1 |
|  |  |  | oxidation-reduction process | 2.36 | 0 |
|  |  |  |  |  |  |
| VI | 48 h, up in both Col-0 and *ein2/jar1* | 2,909 | leucine catabolic process | 8.46 | 5 |
|  |  |  | camalexin biosynthetic process | 6.28 | 8 |
|  |  |  | protein localization to chromosome | 5.92 | 0 |
|  |  |  | chlorophyll catabolic process | 5.75 | 1 |
|  |  |  | cellular response to decreased oxygen levels | 4.76 | 5 |
|  |  |  | jasmonic acid biosynthetic process | 4.52 | 2 |
|  |  |  | regulation of jasmonic acid mediated signaling pathway | 4.42 | 0 |
|  |  |  | defense response to insect | 4.38 | 0 |
|  |  |  | defense response to bacterium, incompatible interaction | 4.19 | 2 |
|  |  |  | trehalose metabolic process | 3.86 | 1 |
|  |  |  | response to absence of light | 3.76 | 0 |
|  |  |  | response to chitin | 3.58 | 2 |
|  |  |  | response to nutrient | 3.52 | 0 |
|  |  |  | tryptophan metabolic process | 3.48 | 2 |
|  |  |  | fatty acid beta-oxidation | 3.44 | 9 |
|  |  |  | autophagosome organization | 3.39 | 3 |
|  |  |  | defense response to fungus, incompatible interaction | 3.29 | 4 |
|  |  |  | response to oomycetes | 3.16 | 0 |
|  |  |  | response to wounding | 3.15 | 0 |
|  |  |  | response to salicylic acid | 3.14 | 1 |
|  |  |  | plant organ senescence | 3.06 | 2 |
|  |  |  | response to virus | 2.96 | 0 |
|  |  |  | response to antibiotic | 2.83 | 4 |
|  |  |  | response to drug | 2.76 | 2 |
|  |  |  | response to jasmonic acid | 2.72 | 0 |
|  |  |  | regulation of response to external stimulus | 2.67 | 0 |
|  |  |  | cofactor catabolic process | 2.65 | 4 |
|  |  |  | ethylene-activated signaling pathway | 2.63 | 3 |
|  |  |  | regulation of response to biotic stimulus | 2.59 | 0 |
|  |  |  | response to water deprivation | 2.53 | 1 |
|  |  |  | auxin metabolic process | 2.47 | 1 |
|  |  |  | response to starvation | 2.39 | 4 |
|  |  |  | response to acid chemical | 2.38 | 3 |
|  |  |  | regulation of defense response | 2.29 | 0 |
|  |  |  | response to carbohydrate | 2.21 | 0 |
|  |  |  | peptidyl-serine modification | 2.14 | 0 |
|  |  |  | cell death | 2.13 | 0 |
|  |  |  |  |  |  |
| VII | 24 h, down only in Col-0 | 58 | cell wall modification | 14.89 | 2 |
|  |  |  | developmental growth involved in morphogenesis | 11.04 | 2 |
|  |  |  | cell growth | 9.76 | 1 |
|  |  |  | cellular developmental process | 5.02 | 0 |
|  |  |  |  |  |  |
| VIII | 48 h, down only in Col-0 | 999 | glucuronoxylan biosynthetic process | 14.09 | 12 |
|  |  |  | xanthophyll metabolic process | 10.06 | 0 |
|  |  |  | lignin catabolic process | 9.39 | 1 |
|  |  |  | plant-type cell wall modification involved in multidimensional cell growth | 7.31 | 6 |
|  |  |  | plant-type secondary cell wall biogenesis | 7.17 | 4 |
|  |  |  | hydrogen peroxide catabolic process | 3.68 | 4 |
|  |  |  |  |  |  |
| IX | 24 h, down only in *ein2/jar1* | 11 | No statistically significant results |  |  |
|  |  |  |  |  |  |
| X | 48 h, down only in *ein2/jar1* | 1,742 | ‘de novo’ UMP biosynthetic process | 16.07 | 28 |
|  |  |  | leading strand elongation | 12.86 | 2 |
|  |  |  | DNA unwinding involved in DNA replication | 12.50 | 3 |
|  |  |  | maturation of LSU-rRNA from tricistronic rRNA transcript | 11.13 | 1 |
|  |  |  | snoRNA 3’-end processing | 10.23 | 2 |
|  |  |  | histidine biosynthetic process | 10.23 | 2 |
|  |  |  | rRNA pseudouridine synthesis | 10.05 | 2 |
|  |  |  | ribosomal large subunit assembly | 9.99 | 1 |
|  |  |  | endonucleolytic cleavage involved in rRNA processing | 9.89 | 5 |
|  |  |  | ribosomal small subunit assembly | 9.82 | 2 |
|  |  |  | rRNA 3’-end processing | 9.18 | 0 |
|  |  |  | ribosomal small subunit biogenesis | 9.06 | 2 |
|  |  |  | U4 snRNA 3’-end processing | 8.77 | 5 |
|  |  |  | geranyl diphosphate biosynthetic process | 8.66 | 1 |
|  |  |  | maturation of 5.8S rRNA from tricistronic rRNA transcript | 8.46 | 3 |
|  |  |  | ‘de novo’ pyrimidine nucleobase biosynthetic process | 8.04 | 1 |
|  |  |  | lysine biosynthetic process via diaminopimelate | 8.04 | 5 |
|  |  |  | arginine biosynthetic process | 8.04 | 2 |
|  |  |  | cytoplasmic translation | 7.81 | 0 |
|  |  |  | rRNA metabolic process | 7.41 | 1 |
|  |  |  | geranylgeranyl disphosphate biosynthetic process | 7.31 | 1 |
|  |  |  | nuclear-transcribed mRNA catabolic process, exonucleolytic, 3’-5’ | 6.89 | 0 |
|  |  |  | nuclear mRNA surveillance | 6.89 | 2 |
|  |  |  | mitochondrial RNA processing | 6.89 | 0 |
|  |  |  | leucine biosynthetic process | 6.89 | 5 |
|  |  |  | mitochondrial gene expression | 6.73 | 1 |
|  |  |  | establishment of protein localization to mitochondrial membrane | 6.70 | 06.70 |
|  |  |  | cytidine to uridine editing | 6.70 | 1 |
|  |  |  | ribonucleoprotein complex assembly | 6.24 | 7 |
|  |  |  | chloroplast fission | 6.12 | 1 |
|  |  |  | mitochondrial RNA metabolic process | 6.06 | 3 |
|  |  |  | translation | 5.91 | 6 |
|  |  |  | protein targeting to mitochondrion | 5.87 | 11 |
|  |  |  | ncRNA processing | 5.83 | 6 |
|  |  |  | DNA replication initiation | 5.56 | 2 |
|  |  |  | glycogen metabolic process | 5.36 | 1 |
|  |  |  | RNA secondary structure unwinding | 5.02 | 0 |
|  |  |  | ‘de novo’ protein folding | 5.02 | 1 |
|  |  |  | embryo sac central cell differentiation | 5.19 | 5 |
|  |  |  | regulation of DNA replication | 4.52 | 0 |
|  |  |  | negative regulation of DNA metabolic process | 4.50 | 1 |
|  |  |  | cell proliferation | 3.76 | 0 |
|  |  |  | histone methylation | 3.40 | 7 |
|  |  |  | nucleocytoplasmic transport | 2.52 | 1 |
|  |  |  | ribonucleoside monophosphate metabolic process | 2.52 | 3 |
|  |  |  | DNA metabolic process | 2.41 | 1 |
|  |  |  | mitotic cell cycle | 2.22 | 0 |
|  |  |  | DNA repair | 2.17 | 1 |
|  |  |  | gene expression | 2.16 | 1 |
|  |  |  | embryo development ending in seed dormancy | 2.03 | 0 |
|  |  |  |  |  |  |
| XI | 24 h, down in both Col-0 and *ein2/jar1* | 17 | No statistically significant results |  |  |
|  |  |  |  |  |  |
| XII | 48 h, down in both Col-0 and *ein2/jar1* | 1,857 | glycine decarboxylation via glycine cleavage system | 12.83 | 6 |
|  |  |  | cell-cell junction assembly | 11.97 | 1 |
|  |  |  | reductive pentose-phosphate cycle | 8.31 | 3 |
|  |  |  | plant-type primary cell wall biogenesis | 7.48 | 4 |
|  |  |  | gluconeogenesis | 5.99 | 4 |
|  |  |  | phloem transport | 5.82 | 1 |
|  |  |  | very long-chain fatty acid biosynthetic process | 5.82 | 3 |
|  |  |  | cell wall pectin metabolic process | 5.70 | 1 |
|  |  |  | negative regulation of actin filament polymerization | 5.61 | 4 |
|  |  |  | suberin biosynthetic process | 5.51 | 2 |
|  |  |  | chaperone cofactor-dependent protein refolding | 5.39 | 3 |
|  |  |  | ATP synthesis coupled proton transport | 5.34 | 1 |
|  |  |  | protein refolding | 5.16 | 0 |
|  |  |  | mucilage metabolic process involved in seed coat development | 4.99 | 0 |
|  |  |  | purine ribonucleoside triphosphate biosynthetic process | 5.16 | 57 |
|  |  |  | ATP hydrolysis coupled proton transport | 4.60 | 11 |
|  |  |  | cellulose biosynthetic process | 4.55 | 6 |
|  |  |  | mucilage metabolic process | 4.53 | 1 |
|  |  |  | purine ribonucleoside biosynthetic process | 4.36 | 8 |
|  |  |  | pectin biosynthetic process | 4.23 | 1 |
|  |  |  | photorespiration | 4.16 | 1 |
|  |  |  | chlorophyll biosynthetic process | 4.01 | 8 |
|  |  |  | pectin metabolic process | 3.87 | 3 |
|  |  |  | response to unfolded protein | 3.74 | 0 |
|  |  |  | tricarboxylic acid metabolic process | 3.61 | 7 |
|  |  |  | response to karrikin | 3.60 | 0 |
|  |  |  | flavonoid biosynthetic process | 3.57 | 1 |
|  |  |  | acyl-CoA metabolic process | 3.29 | 3 |
|  |  |  | plant-type secondary cell wall biogenesis | 3.26 | 3 |
|  |  |  | cellular glucan metabolic process | 3.25 | 8 |
|  |  |  | carbohydrate biosynthetic process | 3.23 | 2 |
|  |  |  | response to cytokinin | 3.14 | 0 |
|  |  |  | carbohydrate catabolic process | 3.13 | 0 |
|  |  |  | phenylpropanoid metabolic process | 3.08 | 2 |
|  |  |  | generation of precursor metabolites and energy | 2.97 | 2 |
|  |  |  | cell wall organization or biogenesis | 2.92 | 3 |
|  |  |  | steroid biosynthetic process | 2.84 | 0 |
|  |  |  | sterol metabolic process | 2.62 | 0 |
|  |  |  | microtubule cytoskeleton organization | 2.59 | 2 |
|  |  |  | response to cadmium ion | 2.47 | 1 |
|  |  |  | rhythmic process | 2.43 | 0 |
|  |  |  | organic hydroxyl compound biosynthetic process | 2.28 | 0 |
|  |  |  | cell growth | 2.03 | 0 |

*The GO term may be a specific subclass or a parent term. The functional class GO terms with fold enrichment >2.00 are listed. When multiple terms are significantly enriched within the same functional class, only the GO term with the highest fold enrichment is listed.

** The number of GO term categories that are related to the listed term (within the same functional class, either as a subclass or parent class) and are also significantly enriched >2.00.

**Supplementary Figures**

**
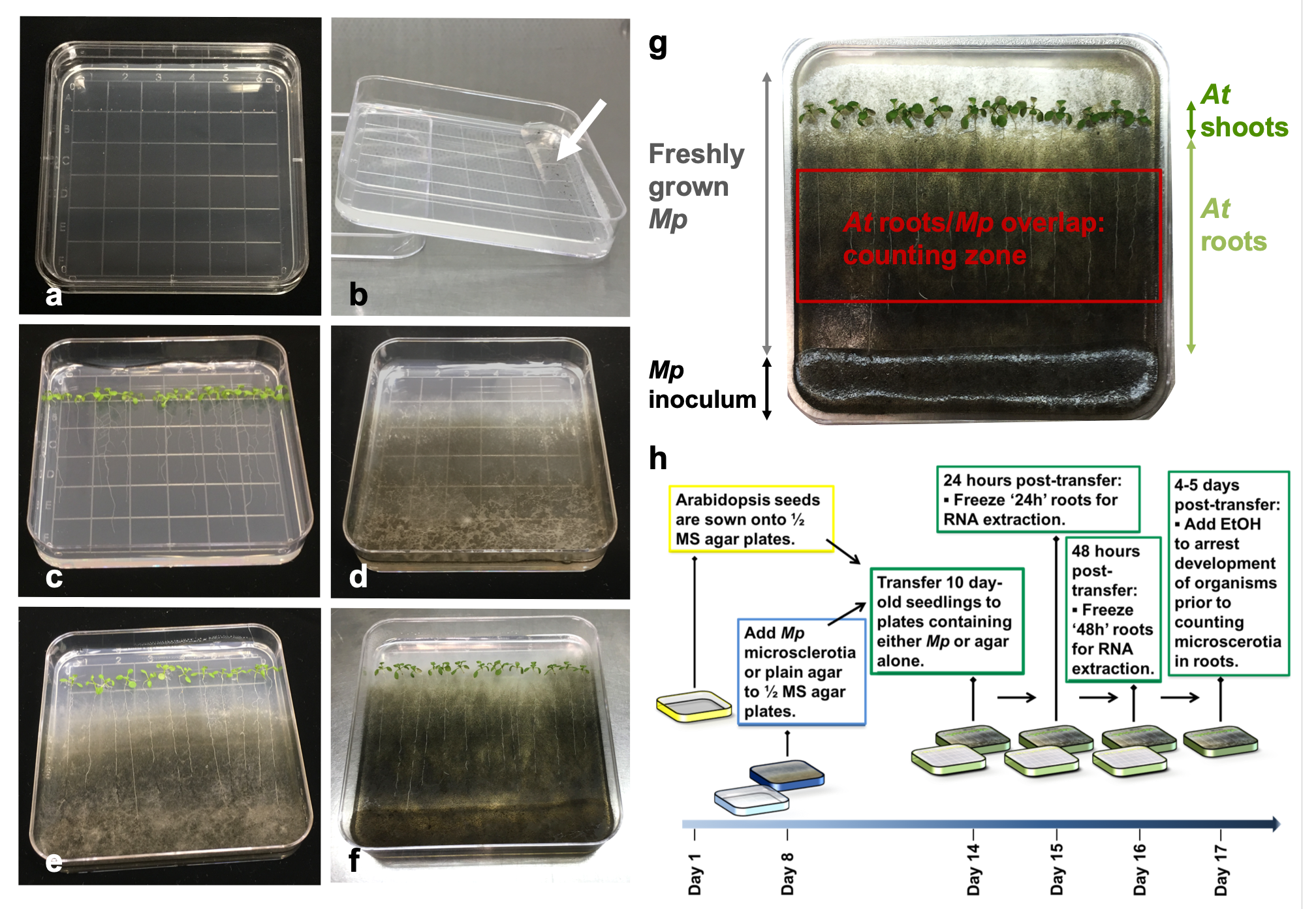
**

**Figure S1.** A plate-based assay system allows for quantitative analysis of *Mp* susceptibility levels in Arabidopsis roots. (a) Arabidopsis seeds are sown on ½ MS agar. (b) *Mp* inoculum, microsclerotia in ½ MS agar, is added to ½ MS agar plates and allowed to grow for six days, forming a *Mp* infection plate (d). (c) Arabidopsis seedlings grown for ten days prior to transfer to treatment plates are shown. (e, f) Arabidopsis seedlings 0.5 hours post transfer (hpt) (e) and 24 hpt to *Mp* infection plates (f). (g) A diagrammed infection plate. (h) Assay timeline.


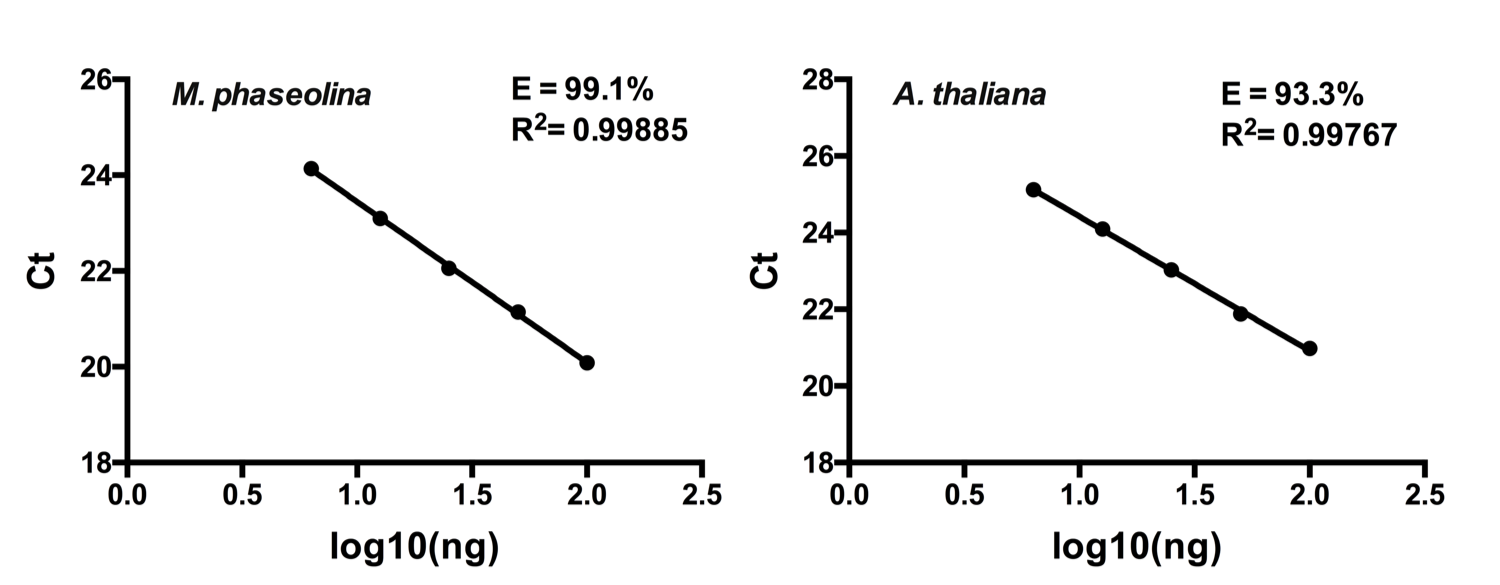


**Figure S2.** SCAR-qPCR standard curves. The ratios of *Mp* and Arabidopsis genomic DNA were calculated by the standard curve method. Serial dilutions of *Mp* and Arabidopsis genomic DNAs were used for standard curve generation. The relative amounts of *Mp* and Arabidopsis genomic DNA were calculated by normalizing *Mp* *MpSyk* to Arabidopsis *AtSK11* measured by qPCR^52^.

**
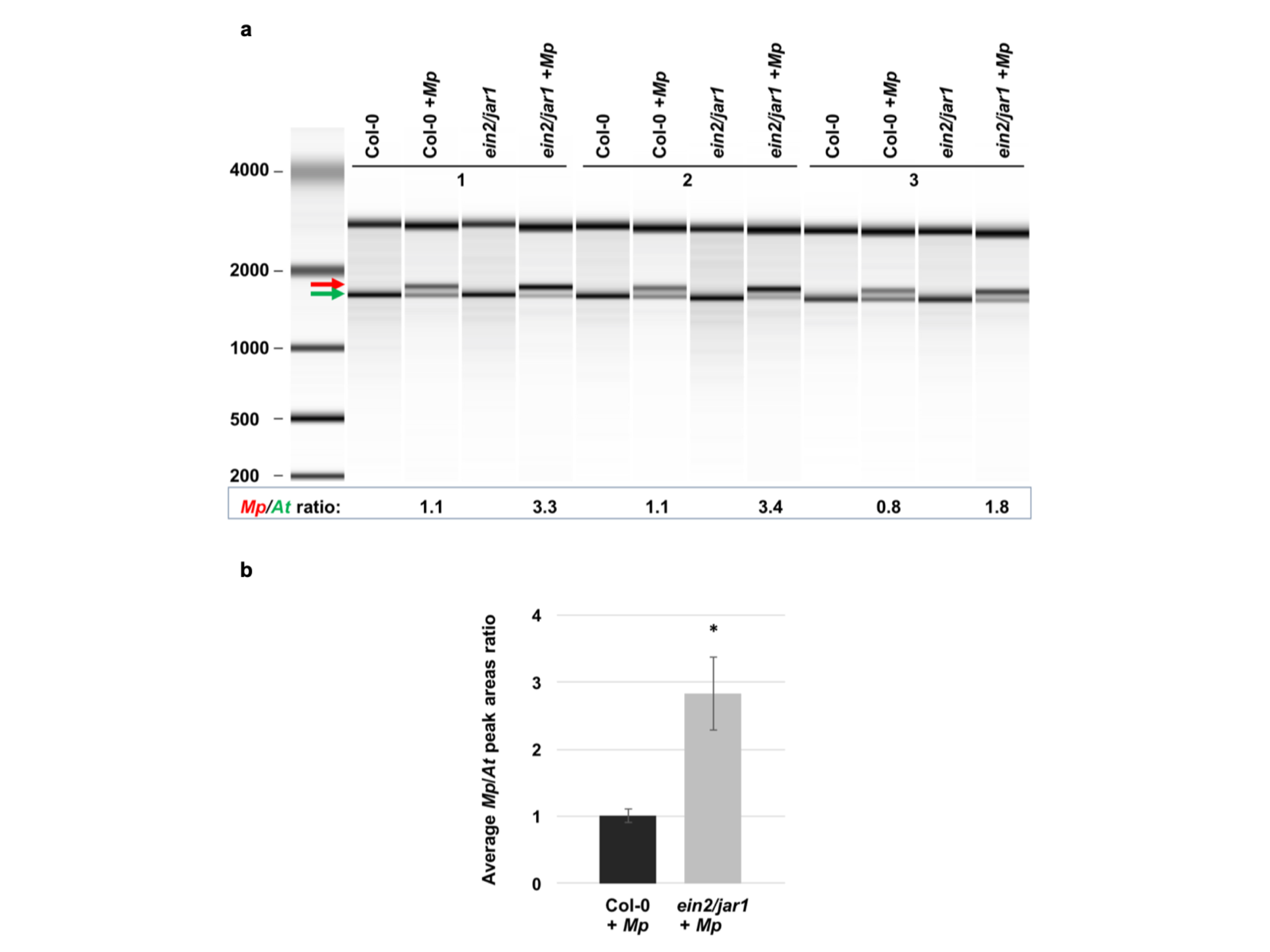
**

**Figure S3.** *ein2/jar1* roots contained more *Mp* RNA than Col-0 roots. (A) Shown is the Bioanalyzer’s 2100 Expert Software-generated artificial gel image representing quantitative RNA data as band intensities in the electrophoresis file run summary of Arabidopsis (*At*) root RNA samples with and without *Mp* 48 hpc. The red arrow points to *Mp* 18S RNA bands. The green arrow points to *At* 18S RNA bands. The ratio of *Mp* 18S to *At* 18S band intensity was significantly higher in *ein2/jar1* than in Col-0, indicating that *Mp* RNA levels were higher in *ein2/jar1* roots than in wild type roots in three (numbered 1, 2 and 3) independent biological replicates. (B) Graph of ratios between *Mp* 18S to *At* 18S bands quantified using ImageJ^99^. Statistical significance relative to Col-0 determined by Student’s t-test, asterisk; *p* < 0.05.

**
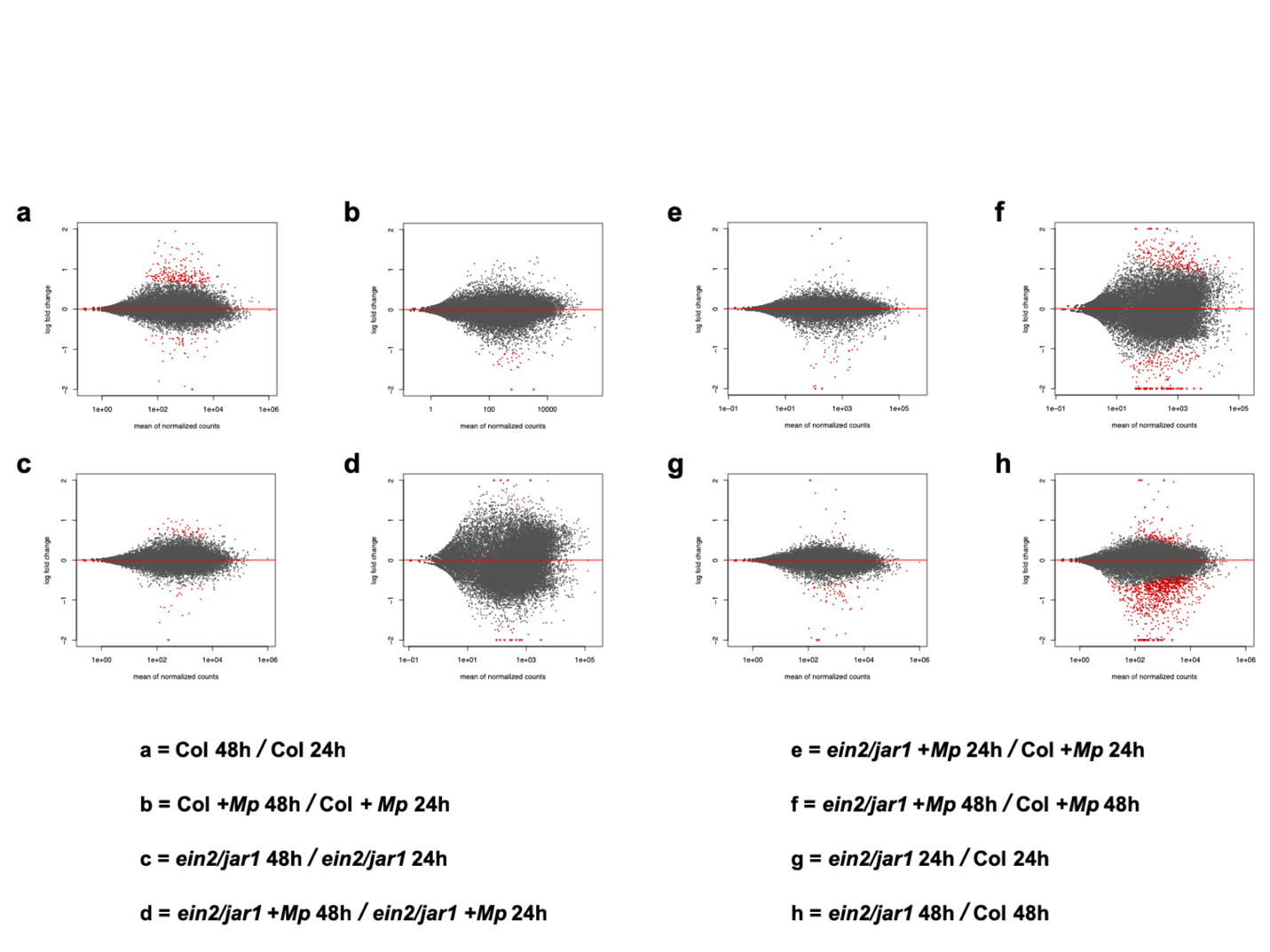
**

**Figure S4.** MA plots portray RNA-seq transcriptome trends. MA plots show the log_2_ fold changes attributable to a given variable over the mean of normalized counts for the compared samples. Genes with similar expression levels in two samples appeared near the horizontal line y = 0. Points which fell out of the window were plotted as open triangles pointing either up or down. Red points, adjusted-*p* < 0.01.
